# Dietary antioxidant nutrients intake and pneumonia mortality in Japanese men and women: the Japan Public Health Center-Based Prospective Study

**DOI:** 10.1265/ehpm.25-00186

**Published:** 2025-09-10

**Authors:** Ling Zha, Tetsuhisa Kitamura, Taiki Yamaji, Motoki Iwasaki, Manami Inoue, Shoichiro Tsugane, Norie Sawada

**Affiliations:** 1 Environmental Medicine and Population Sciences, Graduate School of Medicine, The University of Osaka, Suita, Japan; 2 Division of Epidemiology, Institute for Cancer Control, National Cancer Center, Tokyo, Japan; 3 Division of Cohort Research, Institute for Cancer Control, National Cancer Center, Tokyo, Japan; 4 Division of Prevention, Institute for Cancer Control, National Cancer Center, Tokyo, Japan; 5 Graduate School of Public Health, International University of Health and Welfare, Tokyo, Japan

**Keywords:** Pneumonia mortality, Antioxidant nutrients, Vitamin C, Vitamin E, Cryptoxanthin, Lycopene, Cohort study

## Abstract

**Background:**

Pneumonia is a major global public health concern. Taking antioxidant nutrients has attracted attention for their potential role in reducing pneumonia mortality. Although studies in Western countries have evaluated this association, the current evidence remains controversial, and research in Asia remains limited. This cohort study investigated the association between dietary antioxidant nutrients intake and pneumonia mortality in Japanese population.

**Methods:**

Data were collected from the Japan Public Health Center-based Prospective Study between 1995 and 1998, with follow-up until the end of 2018. The intake of antioxidant nutrients was assessed using a validated food-frequency questionnaire. The Cox proportional hazard model was employed to calculate hazard ratios (HRs) and *p*-trends for pneumonia mortality, adjusting for potential confounding factors including age, area, body mass index, smoking status, alcohol intake, physical activity, postmenopausal status, occupation, coffee intake, green tea intake, antihypertensive medication use, vitamin-supplement use, and total energy intake.

**Results:**

The analysis included data from 39,850 men and 46,705 women. Over a median follow-up of 20.8 years, 813 men and 477 women died from pneumonia. The multivariable model revealed that a higher intake of cryptoxanthin (*p*-trend = 0.027 in men; 0.019 in women), lycopene (*p*-trend = 0.016 in women), vitamin C (*p*-trend = 0.022 in men), and vitamin E (*p*-trend = 0.031 in women) was significantly associated with a reduction in pneumonia mortality.

**Conclusions:**

Higher dietary intake of cryptonxanthin, lycopene, and vitamins C and E was associated with a low risk of pneumonia mortality in Japanese adults.

**Supplementary information:**

The online version contains supplementary material available at https://doi.org/10.1265/ehpm.25-00186.

## Introduction

Pneumonia is a common and serious respiratory infection that remains a leading cause of death worldwide. There has been an increasingly significant global health concern, particularly among vulnerable populations such as children under five and older adults. It caused approximately 2.5 million deaths globally in 2019 [1]. In Japan, pneumonia has persistently been among the top five causes of death since 1975, reflecting its severe impact on public health [2]. In 2019, it ranked as the third most common cause of death among men and the fifth among women, resulting in 53,076 and 42,442 deaths, respectively [3].

Well-known risk factors for pneumonia include advanced age, comorbid conditions, and smoking. However, there has been a growing interest in the role of nutritional status, particularly the intake of antioxidant nutrients, which is a modifiable factor. Antioxidant nutrients are dietary compounds naturally found in vegetables, fruits, and other food sources that can neutralise reactive oxygen species. The major antioxidant nutrients include vitamins A (retinol), C, E, and carotenoids, lycopene, and cryptoxanthin. Vitamins C and E are potent antioxidants that directly neutralize free radicals and reduce oxidative injury [4–9]. Carotenoids such as α-carotene and β-carotene serve as precursors to retinol, which are essential for maintaining respiratory tract integrity and immune function [6, 10, 11], while lycopene and cryptoxanthin exhibit strong antioxidant activities independent of vitamin A synthesis [12, 13]. These nutrients have attracted significant attention as potential preventive factors against pneumonia due to their protective role in protecting lung tissue against oxidative damage and inflammation, which are key processes implicated in the pathogenesis and severity of respiratory infections [4, 14–18].

Previous epidemiological studies, most of which have been conducted in Western countries, have evaluated the association between antioxidant nutrients intake and the risk of pneumonia or respiratory-related mortality; however, the findings have been controversial [4, 8, 9, 11, 19–24]. Some observational studies reported higher serum antioxidant concentrations, including vitamins C and E, and β-carotene, were associated with lower risk of respiratory disease morbidity and mortality [4, 11, 19–21]. However, a meta-analysis on vitamin C supplementation remains has shown limited or uncertain effectiveness for preventing or treating pneumonia in clinical trials [22]. Vitamin E and β-carotene supplementation have demonstrated heterogeneous effects, with beneficial outcomes observed predominantly in certain subgroups, such as older adults, but inconsistent or even adverse effects reported in other populations, notably smokers [8, 9, 21, 23, 24].

Given these inconsistent findings, the evidence regarding the protective effects of antioxidant nutrients on pneumonia-related mortality remains inconclusive. Furthermore, data from Asian populations are particularly limited. Therefore, the present study aimed to investigate whether dietary intake of specific antioxidant nutrients, including vitamin C, vitamin E, α-carotene, β-carotene, retinol, cryptoxanthin, lycopene, as well as vegetables and fruits, is associated with a reduced risk of pneumonia mortality by using data from a prospective population-based cohort study in Japan.

## Methods

### Study design and population

The Japan Public Health Center-based prospective study (JPHC study), launched between 1990 and 1993 and known as the baseline survey, is a prospective, large-scale, population-based cohort study. It included participants aged 40–69 years from 11 public health center areas across Japan [25]. The primary objective of the JPHC study was to determine the association between lifestyle habits and diseases, establishing evidence for the prevention and control of cancer and cardiovascular disease through long-term follow-up [25]. Participants were recruited to complete a self-administered questionnaire on lifestyle and dietary habits. All participants were followed up to obtain data on the occurrence of migration, cancer, cardiovascular disease, and death. The details of the study design have been reported elsewhere [25]. Subsequently, two follow-up surveys were conducted to assess updates in lifestyle and dietary habits: the 5-year survey conducted between 1995 and 1998, and the 10-year survey conducted between 2000 and 2003. Because the food-frequency questionnaire (FFQ) used in the 5-year survey was more comprehensive than that in the baseline survey and had been validated in previous studies, we used the information of the 5-year survey as the starting point in our present study [26–28].

A total of 103,215 participants who completed the 5-year survey and met the eligibility criteria were initially enrolled in our study. We excluded 11,076 participants who self-reported a history of severe disease, including cancer, stroke, ischemic heart disease, renal disease, or diabetes requiring medication at the baseline survey or the 5-year survey. Additionally, 5,553 participants were excluded due to missing data or extreme values (lower and upper 2.5th percentile) of total energy intake, and 31 participants were lost to follow-up. Consequently, data from 86,555 participants (39,850 men and 46,705 women) were eligible for analysis. The study was approved by the institutional review board of the National Cancer Center, Japan (13-021), and The University of Osaka (14020). The participants were informed of the study objectives, and those who completed the survey questionnaire were regarded as having consented to participation.

### Ascertainment of antioxidant nutrients intake

In the present study, the dietary intake of antioxidant nutrients including vitamin C, vitamin E (α-, β-, γ-, and δ-tocopherols), α-carotene, β-carotene, retinol, cryptoxanthin, lycopene, as well as vegetables, fruits, and green and yellow vegetables, was examined as exposures. Vitamin supplementation was not included as exposure in the study, as the focus was specifically on antioxidant nutrients obtained through dietary sources. The FFQ used in the 5-year survey covered dietary information on the frequency and portion size of 147 food and beverage items consumed in the past one year. Food frequency was classified into nine categories: never, one to three times/month, one to two times/week, three to four times/week, five to six times/week, once/day, two to three times/day, four to six times/day, and seven times/day. The portion size consumed was classified into three categories: 50% smaller than the standard, same as the standard, and 50% larger than the standard portion size. The daily consumption of each food item was calculated by multiplying the frequency by the relative portion size. The daily consumption of each nutrient was calculated by multiplying the frequency of consuming each food by its nutrient content per serving. The sum of nutrient intakes from all food items was then computed. Nutrient calculations were based on values from the Standard Tables of Food Composition in Japan (7th edition), using the same food groups for our analyses [29]. The validity and reproducibility of this FFQ were verified by using 28- or 14-day dietary records as the gold standard. The dietary records were collected from cohort subsamples, and the estimated dietary intakes from the FFQ were compared to those obtained from the dietary records [26, 28, 30]. The Spearman’s rank correlation coefficients between dietary records and antioxidant nutrients estimated from the FFQ were moderate, ranging from 0.37 for retinol to 0.51 for α-carotene in men, and from 0.29 for cryptoxanthin to 0.53 for α-carotene in women [26, 28, 30].

### Follow-up and outcome identification

Participants were followed from the date of response to the 5-year survey until censored on the date of death, date of migration out of Japan or the end of follow-up (December 31, 2018 for 9 areas, December 31, 2009 for 1 area, and December 31, 2012 for 1 area), whichever occurred first. Death certificates were used with the permission of the Ministry of Health, Labor, and Welfare to confirm the cause of death with the International Statistical Classification of Diseases and Related Health Problems, Tenth Revision. Codes J10.0, J11.0, and J12–J18 were used to identify pneumonia cases.

### Statistical analysis

Participants were divided into quintiles based on their energy-adjusted intake of vitamin C, vitamin E, α-carotene, β-carotene, retinol, cryptoxanthin, lycopene, vegetables, and fruits, stratified by gender. All nutritional covariates, except for alcohol, coffee, and green tea, were adjusted for total energy intake using the residual method [31]. Both univariable and multivariable Cox proportional hazard regression models were performed to estimate hazard ratios (HRs), 95% confidence intervals (CIs), and *p*-trends for pneumonia mortality across quintiles of antioxidant nutrient intake, with the lowest quintile as the reference category. In the multivariable models, model 1 was adjusted for age at the 5-year survey (continuous) and public health center area (11 areas). Model 2 was further adjusted for body mass index (BMI kg/m^2^; 14 to <21, 21 to <23, 23 to <25, or 25 to 40), smoking status (never, past, current with 1 to 19, or current with ≥20 cigarettes/day), alcohol intake (nondrinker, occasional drinker, <300, or ≥300 g/week in men; nondrinker, occasional drinker, <150, or ≥150 g/week in women), physical activity (metabolic equivalents [METs]; quartiles), occupation (salaried/self-employed/professional, agriculture/forestry/fishery, housework/unemployed, multiple occupations/others), frequency of coffee consumption (never, 1 to 6 cups/week, 1 cup/day, 2 to 3 cups/day, or ≥4 cups/day), frequency of green tea consumption (never, 1 to 6 cups/week, 1 cup/day, 2 to 3 cups/day, or ≥4 cups/day), use of medication for hypertension (yes or no), use of vitamin supplementation (yes or no), postmenopausal status (only women; yes or no), and total energy intake (continuous). These potential confounders were selected based on previous studies [32–34]. A sensitivity analysis was conducted with model 2 by excluding participants who died from pneumonia within the first three years of follow-up. This approach was taken to minimize the impact of potential bias from participants who may have had preexisting conditions leading to pneumonia. We also conducted additional sensitivity analyses, including both multivariable and complete-case analyses, excluding participants who reported using vitamin supplements at the 5-year survey. Furthermore, stratified analyses by age (≤60 or >60 years), BMI (≤median or >median) and smoking status (never or ever smoking) were conducted based on the previous studies from the JPHC study, and the *p*-value for interaction was calculated. These analyses were based on the hypothesis that the effect of antioxidant nutrients intake on pneumonia mortality may vary between older and younger participants, those with lower versus higher BMI, and nonsmokers versus ever smokers. Since over 80% of the women were never smokers, the stratified analysis for women was conducted only among never smokers.

Multiple imputation was performed to address missing data for covariates used in model 2 by chained equations, and 20 rounds of imputation were performed. Final estimates were calculated according to Rubin’s rule [35]. Missing data included BMI for 872 (2.2%) men and 1,225 (2.6%) women, smoking status for 595 (1.5%) men and 2,764 (5.6%) women, alcohol intake for 420 (1.1%) men and 1,286 (2.8%) women, physical exercise for 6,753 (16.9%) men and 8,093 (17.3%) women, coffee intake for 1,409 (3.5%) men and 1,797 (3.8%) women, green tea intake for 631 (1.6%) men and 1,711 (1.4%) women, menopausal status for 897 (2.2%) women, and occupation for 1,455 (3.7%) men and 1,663 (3.6%) women. Furthermore, a complete case analysis was conducted in addition to the results obtained by using the imputation method. The *p*-values for all of the tests were two-sided and significance level was set as *p*-value < 0.05. All statistical analyses were conducted using Stata MP 18.0 (StataCorp, College Station, TX, USA).

## Results

During a median follow-up of 20.8 years (Interquartile range: 15.8–23.7 years), a total of 813 men and 477 women died from pneumonia. Table 1 presents the baseline characteristics of participants from the 5-year survey conducted between 1995 and 1998, divided by quintile according to their dietary intake of vitamin C. Baseline characteristics for other antioxidant nutrients, vegetables, and fruits are showed in Supplementary Table 1. Men and women with higher intakes of these nutrients were generally older, smoked less, consumed less alcohol and coffee, and drank more green tea. They also reported a higher prevalence of hypertension requiring medication and were more likely to consume more fish and less red meat.

**Table 1:** Basic Characteristics by Quintiles of Dietary Intake of Vitamin C in Japanese Men and Women.

	**Men (N = 39,850)**					

	**Q1**	**Q2**	**Q3**	**Q4**	**Q5**	***P*-value**
Number of participants	7970	7970	7970	7970	7970	
Age (years), mean (SD)^a^	54.1 (7.2)	54.9 (7.4)	56.1 (7.6)	57.3 (7.7)	59.1 (8.0)	<0.001
BMI (kg/m^2^), mean (SD)^a^	23.6 (3.0)	23.6 (2.9)	23.5 (2.9)	23.6 (2.8)	23.6 (2.8)	0.122
Current smokers, %^b^	4468 (56.1%)	3960 (49.7%)	3535 (44.4%)	3301 (41.4%)	2842 (35.7%)	<0.001
Alcohol intake (≥1 day/week), %^b^	6331 (79.4%)	5943 (74.6%)	5574 (69.9%)	5038 (63.2%)	4271 (53.6%)	<0.001
METs, median (IQR)^c^	33.7 (27.1, 42.7)	31.9 (27.1, 42.7)	33.7 (27.1, 42.7)	33.7 (27.1, 42.7)	33.7 (27.1, 42.7)	0.865
Medication for hypertension (yes), %^b^	1261 (15.8%)	1394 (17.5%)	1469 (18.4%)	1543 (19.4%)	1704 (21.4%)	<0.001
Vitamin supplement (yes), %^b^	361 (4.5%)	436 (5.5%)	465 (5.8%)	515 (6.5%)	556 (7.0%)	<0.001
Postmenopausal, %^b^						
Occupation						<0.001
Salaried/self-employed/professional	4695 (62.1%)	4712 (61.2%)	4449 (57.6%)	4259 (55.1%)	3712 (48.3%)	
Agriculture/forestry/fishery	1150 (15.2%)	1201 (15.6%)	1327 (17.2%)	1423 (18.4%)	1761 (22.9%)	
Housework/unemployed	448 (5.9%)	514 (6.7%)	693 (9.0%)	801 (10.4%)	980 (12.7%)	
Multiple occupations/others	1265 (16.7%)	1274 (16.5%)	1250 (16.2%)	1244 (16.1%)	1237 (16.1%)	
Coffee intake (>1 time/day), %^b^	2011 (25.2%)	1967 (24.7%)	1721 (21.6%)	1576 (19.8%)	1260 (15.8%)	<0.001
Green tea intake (>1 time/day), %^b^	2407 (30.2%)	4168 (52.3%)	5285 (66.3%)	5947 (74.6%)	6715 (84.3%)	<0.001
Total energy intake (kcal/day), median (IQR)^c^	2078.7 (1684.6, 2559.7)	2117.8 (1721.4, 2577.8)	2112.0 (1722.4, 2584.0)	2101.5 (1713.4, 2559.9)	2035.5 (1661.9, 2493.1)	<0.001
Fish (g/day), median (IQR)^c^	61.1 (38.9, 92.5)	75.8 (51.4, 108.1)	81.0 (56.4, 114.4)	84.6 (58.4, 118.3)	84.4 (57.3, 119.7)	<0.001
Meat (g/day), median (IQR)^c^	51.6 (30.1, 81.0)	57.5 (35.7, 85.6)	56.5 (35.7, 84.6)	55.2 (35.6, 80.1)	49.0 (30.0, 72.8)	<0.001
Vegetables (g/day), median (IQR)^c^	85.1 (56.0, 117.8)	134.5 (101.0, 171.5)	172.1 (129.2, 217.9)	211.0 (157.4, 272.8)	279.2 (200.7, 382.0)	<0.001
Fruits (g/day), median (IQR)^c^	46.5 (21.8, 84.6)	102.3 (59.8, 148.1)	141.5 (91.4, 195.8)	192.5 (128.8, 261.4)	290.1 (189.0, 415.5)	<0.001
Green and yellow vegetables (g/day), median (IQR)^c^	31.9 (18.0, 52.7)	56.4 (36.0, 81.3)	72.7 (48.4, 102.7)	90.6 (61.1, 126.6)	122.2 (80.1, 176.8)	<0.001
Vitamin C (mg/day), median (IQR)^c^	47.1 (35.7, 55.5)	75.8 (69.2, 82.2)	102.2 (95.2, 109.4)	135.1 (125.5, 145.5)	197.1 (174.7, 234.4)	<0.001

	**Women (N = 46,705)**					

	**Q1**	**Q2**	**Q3**	**Q4**	**Q5**	***P*-value**

Number of participants	9341	9341	9341	9341	9341	
Age (years), mean (SD)^a^	55.2 (7.8)	55.9 (7.8)	56.6 (7.8)	57.6 (7.9)	58.3 (7.8)	<0.001
BMI (kg/m^2^), mean (SD)^a^	23.6 (3.3)	23.4 (3.1)	23.4 (3.2)	23.3 (3.1)	23.4 (3.1)	<0.001
Current smokers, %^b^	839 (9.0%)	546 (5.8%)	467 (5.0%)	370 (4.0%)	372 (4.0%)	<0.001
Alcohol intake (≥1 day/week), %^b^	1658 (17.7%)	1409 (15.1%)	1243 (13.3%)	1072 (11.5%)	885 (9.5%)	<0.001
METs, median (IQR)^c^	31.9 (27.1, 34.2)	31.9 (27.1, 34.2)	31.9 (27.1, 34.2)	31.9 (27.1, 34.2)	31.9 (27.1, 34.2)	<0.001
Medication for hypertension (yes), %^b^	1648 (17.6%)	1713 (18.3%)	1935 (20.7%)	1946 (20.8%)	2057 (22.0%)	<0.001
Vitamin supplement (yes), %^b^	820 (8.8%)	956 (10.2%)	1046 (11.2%)	1055 (11.3%)	988 (10.6%)	<0.001
Postmenopausal, %^b^	6295 (69.8%)	6654 (73.0%)	6998 (76.3%)	7350 (80.0%)	7642 (83.2%)	<0.001
Occupation						<0.001
Salaried/self-employed/professional	3085 (35.0%)	3028 (33.5%)	2763 (30.6%)	2485 (27.3%)	2260 (24.9%)	
Agriculture/forestry/fishery	1209 (13.7%)	1130 (12.5%)	1049 (11.6%)	1080 (11.9%)	1071 (11.8%)	
Housework/unemployed	2410 (27.3%)	2677 (29.6%)	2967 (32.8%)	3246 (35.7%)	3494 (38.6%)	
Multiple occupations/others	2111 (23.9%)	2202 (24.4%)	2260 (25.0%)	2281 (25.1%)	2234 (24.7%)	
Coffee intake (>1 time/day), %^b^	2209 (23.6%)	1914 (20.5%)	1708 (18.3%)	1404 (15.0%)	1149 (12.3%)	<0.001
Green tea intake (>1 time/day), %^b^	3523 (37.7%)	5704 (61.1%)	6800 (72.8%)	7615 (81.5%)	8243 (88.2%)	<0.001
Total energy intake (kcal/day), median (IQR)^c^	1760.8 (1402.0, 2230.0)	1781.1 (1457.0, 2201.7)	1794.0 (1476.7, 2198.4)	1810.5 (1494.7, 2192.0)	1747.8 (1425.0, 2140.0)	<0.001
Fish (g/day), median (IQR)^c^	63.7 (40.8, 94.7)	76.1 (52.4, 106.6)	80.0 (56.5, 110.0)	80.6 (55.7, 110.8)	76.5 (52.8, 105.9)	<0.001
Meat (g/day), median (IQR)^c^	55.8 (33.0, 86.6)	53.3 (33.3, 78.0)	49.4 (31.9, 71.5)	44.6 (28.4, 64.4)	38.4 (23.1, 57.6)	<0.001
Vegetables (g/day), median (IQR)^c^	117.0 (82.6, 155.3)	170.9 (132.6, 216.5)	204.8 (157.2, 261.4)	241.5 (183.3, 310.7)	301.5 (216.6, 407.7)	<0.001
Fruits (g/day), median (IQR)^c^	92.5 (50.9, 140.3)	156.4 (110.3, 209.4)	204.8 (148.4, 266.0)	258.7 (187.0, 338.3)	380.3 (266.5, 512.3)	<0.001
Green and yellow vegetables (g/day), median (IQR)^c^	46.4 (29.4, 69.2)	71.5 (50.2, 100.4)	88.0 (60.9, 121.8)	105.9 (73.0, 145.5)	131.3 (86.0, 189.0)	<0.001
Vitamin C (mg/day), median (IQR)^c^	70.1 (56.5, 80.4)	105.3 (97.5, 112.6)	135.1 (127.5, 142.8)	171.2 (161.0, 183.0)	235.2 (212.8, 272.8)	<0.001

Abbreviations: SD, standard deviation; BMI, body mass index; METs, metabolic equivalents; IQR, interquartile range.

Detailed participant characteristics for vitamin E, α-carotene, β-carotene, cryptoxanthin, lycopene, vegetables, fruits, and green and yellow vegetables are presented in Supplementary Table 1.

^a^*P* values were calculated with ANOVA.

^b^*P* values were calculated with the chi-square test.

^c^*P* values were calculated with the Kruskal-Wallis test.

HRs and 95% CIs of pneumonia mortality according to quintiles of antioxidant nutrients, vegetables, and fruits by gender are shown in Table 2. In men, crude HRs for pneumonia mortality in the highest quintile of vitamin C, vitamin E, and cryptoxanthin intake suggested an increased risk. However, these associations were reversed after adjustment for potential confounding factors in the multivariable models. In the multivariable-adjusted model 2, a significant inverse association was observed between higher intake of cryptoxanthin and reduced pneumonia mortality risk in both genders (*p*-trend = 0.027 in men; *p*-trend = 0.019 in women). Higher intake of lycopene was significantly associated with reduced risk of pneumonia mortality in women, with a *p*-trend of 0.016. In men, the HRs in the second, third, and fifth quintiles of lycopene intake were significantly lower than in the first quintile (Q1). Furthermore, higher intake of vitamin C was significantly associated with reduced pneumonia mortality risk in men (*p*-trend = 0.022), while higher intake of vitamin E showed a similar association in women (*p*-trend = 0.031). In addition, higher consumption of fruits was significantly associated with a reduced risk of pneumonia mortality in both genders (*p*-trend = 0.049 in men; *p*-trend = 0.006 in women). Higher consumption of green and yellow vegetables was significantly associated with a reduced risk of pneumonia mortality in men (*p*-trend = 0.035). The sensitivity analysis, which excluded deaths within the first 3 years of follow-up, confirmed that the inverse associations observed in the main analysis were robust (as shown in Supplementary Table 2). In the complete case analysis, the results for vitamins C and E were consistent with those of the imputation models. For cryptoxanthin in men, the results were similar to the imputation models, although the *p*-trend was marginally nonsignificant in model 2 (as shown in Supplementary Table 4).

**Table 2:** Hazard Ratios for Pneumonia Mortality by Quintiles of Intake of Antioxidant Nutrients, Vegetables, and Fruits.

	**Men (N = 39,850)**					

	**Q1 (Reference)**	**Q2**	**Q3**	**Q4**	**Q5**	***P*-trend**
Vitamin C						
Person-years	145,294	146,626	146,658	146,629	144,531	
Number of cases	134	153	153	175	198	
Crude rate (per 100,000 person-years)	92.23	104.35	104.32	119.35	136.99	
Crude HR	1.00	1.12 (0.89–1.41)	1.12 (0.89–1.41)	1.28 (1.02–1.60)	1.48 (1.19–1.84)	*<0.001*
HR1^a^	1.00	1.00 (0.80–1.27)	0.83 (0.66–1.05)	0.81 (0.64–1.01)	0.73 (0.58–0.92)	*0.001*
HR2^b^	1.00	1.04 (0.82–1.32)	0.88 (0.69–1.12)	0.86 (0.68–1.11)	0.78 (0.61–1.01)	*0.022*
Vitamin E						
Person-years	144,551	146,865	146,508	146,660	145,154	
Number of cases	161	153	123	168	208	
Crude rate (per 100,000 person-years)	111.38	104.18	83.95	114.55	143.30	
Crude HR	1.00	0.93 (0.74–1.15)	0.75 (0.59–0.94)	1.01 (0.82–1.26)	1.28 (1.04–1.57)	*0.009*
HR1^a^	1.00	0.86 (0.69–1.07)	0.61 (0.48–0.77)	0.77 (0.62–0.96)	0.79 (0.63–0.97)	*0.037*
HR2^b^	1.00	0.89 (0.71–1.11)	0.64 (0.50–0.81)	0.84 (0.67–1.06)	0.85 (0.67–1.06)	*0.225*
α-carotene						
Person-years	144,222	146,435	146,220	146,973	145,888	
Number of cases	161	141	166	152	193	
Crude rate (per 100,000 person-years)	111.63	96.29	113.53	103.42	132.29	
Crude HR	1.00	0.86 (0.68–1.08)	1.01 (0.82–1.26)	0.92 (0.73–1.14)	1.18 (0.95–1.45)	*0.095*
HR1^a^	1.00	0.82 (0.66–1.03)	0.91 (0.73–1.13)	0.77 (0.62–0.97)	0.84 (0.68–1.04)	*0.103*
HR2^b^	1.00	0.86 (0.69–1.09)	0.94 (0.76–1.18)	0.82 (0.65–1.03)	0.88 (0.71–1.10)	*0.259*
β-carotene						
Person-years	144,575	145,742	146,906	146,257	146,258	
Number of cases	157	124	161	172	199	
Crude rate (per 100,000 person-years)	108.59	85.08	109.59	117.60	136.06	
Crude HR	1.00	0.78 (0.61–0.98)	1.00 (0.80–1.24)	1.07 (0.86–1.32)	1.23 (1.00–1.52)	*0.003*
HR1^a^	1.00	0.69 (0.55–0.87)	0.79 (0.63–0.98)	0.76 (0.61–0.94)	0.74 (0.60–0.92)	*0.050*
HR2^b^	1.00	0.72 (0.57–0.92)	0.84 (0.67–1.05)	0.81 (0.65–1.01)	0.80 (0.64–1.00)	*0.205*
Retinol						
Person-years	147,720	145,694	146,367	146,559	143,398	
Number of cases	182	167	133	174	157	
Crude rate (per 100,000 person-years)	123.21	114.62	90.87	118.72	109.49	
Crude HR	1.00	0.95 (0.77–1.17)	0.75 (0.60–0.93)	0.97 (0.79–1.20)	0.91 (0.73–1.12)	*0.482*
HR1^a^	1.00	0.88 (0.71–1.08)	0.74 (0.59–0.92)	1.00 (0.81–1.24)	0.84 (0.68–1.05)	*0.382*
HR2^b^	1.00	0.91 (0.74–1.13)	0.77 (0.61–0.96)	1.03 (0.83–1.28)	0.81 (0.65–1.01)	*0.232*
Cryptoxanthin						
Person-years	144,828	147,614	147,773	147,297	142,226	
Number of cases	153	161	159	151	189	
Crude rate (per 100,000 person-years)	105.64	109.07	107.60	102.51	132.89	
Crude HR	1.00	1.01 (0.81–1.27)	1.00 (0.80–1.24)	0.95 (0.76–1.19)	1.27 (1.02–1.57)	*0.080*
HR1^a^	1.00	0.96 (0.77–1.20)	0.86 (0.69–1.08)	0.72 (0.57–0.90)	0.75 (0.60–0.93)	*0.001*
HR2^b^	1.00	1.02 (0.81–1.27)	0.96 (0.76–1.20)	0.81 (0.64–1.02)	0.84 (0.67–1.05)	*0.027*
Lycopene						
Person-years	146,602	145,805	144,334	148,407	144,590	
Number of cases	200	142	168	157	146	
Crude rate (per 100,000 person-years)	136.42	97.39	116.40	105.79	100.98	
Crude HR	1.00	0.72 (0.58–0.90)	0.87 (0.71–1.07)	0.77 (0.63–0.95)	0.75 (0.61–0.93)	*0.026*
HR1^a^	1.00	0.73 (0.59–0.90)	0.77 (0.62–0.94)	0.78 (0.63–0.96)	0.74 (0.60–0.92)	*0.022*
HR2^b^	1.00	0.75 (0.60–0.93)	0.80 (0.65–0.98)	0.88 (0.71–1.09)	0.77 (0.62–0.96)	*0.116*
Vegetables						
Person-years	144,319	145,582	146,048	147,422	146,367	
Number of cases	148	145	151	174	195	
Crude rate (per 100,000 person-years)	102.55	99.60	103.39	118.03	133.23	
Crude HR	1.00	0.96 (0.76–1.21)	0.99 (0.79–1.25)	1.13 (0.90–1.40)	1.27 (1.03–1.57)	0.008
HR1^a^	1.00	0.90 (0.72–1.13)	0.84 (0.67–1.05)	0.85 (0.69–1.07)	0.84 (0.68–1.05)	*0.138*
HR2^b^	1.00	0.95 (0.76–1.20)	0.90 (0.72–1.14)	0.92 (0.74–1.15)	0.90 (0.72–1.13)	*0.368*
Fruits						
Person-years	144,381	147,209	147,411	146,642	144,096	
Number of cases	164	156	144	165	184	
Crude rate (per 100,000 person-years)	113.59	105.97	97.69	112.52	127.69	
Crude HR	1.00	0.92 (0.74–1.14)	0.84 (0.67–1.06)	0.97 (0.78–1.21)	1.12 (0.90–1.38)	*0.233*
HR1^a^	1.00	0.81 (0.65–1.00)	0.71 (0.56–0.88)	0.73 (0.59–0.91)	0.69 (0.56–0.86)	*0.001*
HR2^b^	1.00	0.86 (0.69–1.07)	0.79 (0.63–0.99)	0.82 (0.66–1.03)	0.78 (0.62–0.98)	*0.049*
Green and yellow vegetables						
Person-years	144,004	145,179	147,007	146,897	146,651	
Number of cases	180	142	141	173	177	
Crude rate (per 100,000 person-years)	125.00	97.81	95.91	117.77	120.69	
Crude HR	1.00	0.78 (0.62–0.97)	0.75 (0.60–0.94)	0.92 (0.75–1.14)	0.94 (0.77–1.16)	*0.857*
HR1^a^	1.00	0.74 (0.60–0.93)	0.69 (0.55–0.86)	0.76 (0.62–0.94)	0.71 (0.58–0.88)	*0.007*
HR2^b^	1.00	0.78 (0.62–0.97)	0.73 (0.58–0.91)	0.82 (0.66–1.02)	0.75 (0.60–0.93)	*0.035*

	**Women (N = 46,705)**					

	**Q1 (Reference)**	**Q2**	**Q3**	**Q4**	**Q5**	***P*-trend**

Vitamin C						
Person-years	182,906	183,598	182,886	182,666	181,496	
Number of cases	102	88	89	102	96	
Crude rate (per 100,000 person-years)	55.77	47.93	48.66	55.84	52.89	
Crude HR	1.00	0.86 (0.65–1.14)	0.88 (0.66–1.16)	1.00 (0.76–1.32)	0.95 (0.72–1.26)	*0.863*
HR1^a^	1.00	0.80 (0.60–1.06)	0.74 (0.56–0.99)	0.77 (0.58–1.02)	0.67 (0.50–0.89)	*0.011*
HR2^b^	1.00	0.81 (0.61–1.09)	0.78 (0.58–1.06)	0.82 (0.61–1.10)	0.71 (0.52–0.97)	*0.069*
Vitamin E						
Person-years	178,993	181,729	183,562	184,849	184,420	
Number of cases	120	94	81	95	87	
Crude rate (per 100,000 person-years)	67.04	51.73	44.13	51.39	47.17	
Crude HR	1.00	0.76 (0.58–1.00)	0.65 (0.49–0.86)	0.75 (0.57–0.98)	0.69 (0.52–0.90)	*0.011*
HR1^a^	1.00	0.85 (0.65–1.12)	0.74 (0.56–0.99)	0.85 (0.64–1.12)	0.72 (0.54–0.96)	*0.039*
HR2^b^	1.00	0.87 (0.66–1.14)	0.76 (0.57–1.01)	0.85 (0.64–1.12)	0.71 (0.53–0.95)	*0.031*
α-carotene						
Person-years	180,780	181,975	183,478	183,501	183,819	
Number of cases	106	94	93	86	98	
Crude rate (per 100,000 person-years)	58.63	51.66	50.69	46.87	53.31	
Crude HR	1.00	0.88 (0.67–1.16)	0.86 (0.65–1.13)	0.79 (0.60–1.05)	0.90 (0.68–1.18)	*0.308*
HR1^a^	1.00	1.06 (0.80–1.39)	0.99 (0.75–1.31)	0.97 (0.73–1.30)	0.97 (0.73–1.29)	*0.690*
HR2^b^	1.00	1.06 (0.80–1.40)	1.01 (0.76–1.34)	0.99 (0.74–1.31)	0.97 (0.73–1.29)	*0.707*
β-carotene						
Person-years	179,779	181,830	183,164	184,358	184,422	
Number of cases	102	97	84	89	105	
Crude rate (per 100,000 person-years)	56.74	53.35	45.86	48.28	56.93	
Crude HR	1.00	0.93 (0.71–1.23)	0.80 (0.60–1.07)	0.83 (0.63–1.11)	0.98 (0.75–1.29)	*0.660*
HR1^a^	1.00	0.99 (0.75–1.31)	0.85 (0.63–1.13)	0.85 (0.64–1.13)	0.88 (0.67–1.16)	*0.223*
HR2^b^	1.00	1.01 (0.76–1.33)	0.86 (0.64–1.15)	0.86 (0.65–1.15)	0.89 (0.67–1.18)	*0.242*
Retinol						
Person-years	184,615	181,814	180,072	183,560	183,491	
Number of cases	107	91	97	89	93	
Crude rate (per 100,000 person-years)	57.96	50.05	53.87	48.49	50.68	
Crude HR	1.00	0.88 (0.66–1.16)	0.95 (0.73–1.26)	0.84 (0.64–1.12)	0.88 (0.67–1.16)	*0.347*
HR1^a^	1.00	0.94 (0.71–1.25)	1.08 (0.82–1.42)	1.00 (0.75–1.33)	1.01 (0.76–1.34)	*0.819*
HR2^b^	1.00	0.97 (0.73–1.29)	1.13 (0.85–1.49)	1.06 (0.79–1.41)	1.03 (0.78–1.37)	*0.654*
Cryptoxanthin						
Person-years	181,890	184,934	184,904	182,805	179,020	
Number of cases	112	75	85	92	113	
Crude rate (per 100,000 person-years)	61.58	40.56	45.97	50.33	63.12	
Crude HR	1.00	0.65 (0.49–0.87)	0.74 (0.56–0.98)	0.81 (0.62–1.07)	1.03 (0.80–1.34)	*0.411*
HR1^a^	1.00	0.66 (0.49–0.88)	0.66 (0.50–0.88)	0.63 (0.47–0.83)	0.67 (0.51–0.88)	*0.008*
HR2^b^	1.00	0.67 (0.50–0.90)	0.68 (0.51–0.90)	0.65 (0.49–0.86)	0.70 (0.53–0.92)	*0.019*
Lycopene						
Person-years	184,502	181,982	181,404	184,567	181,097	
Number of cases	128	84	94	89	82	
Crude rate (per 100,000 person-years)	69.38	46.16	51.82	48.22	45.28	
Crude HR	1.00	0.68 (0.51–0.89)	0.76 (0.58–0.99)	0.70 (0.53–0.91)	0.66 (0.50–0.88)	*0.008*
HR1^a^	1.00	0.77 (0.58–1.01)	0.81 (0.62–1.06)	0.74 (0.56–0.97)	0.69 (0.52–0.91)	*0.011*
HR2^b^	1.00	0.78 (0.59–1.03)	0.82 (0.62–1.07)	0.77 (0.58–1.01)	0.70 (0.53–0.92)	*0.016*
Vegetables						
Person-years	178,606	181,541	182,154	184,527	186,724	
Number of cases	108	81	99	89	100	
Crude rate (per 100,000 person-years)	60.47	44.62	54.35	48.23	53.56	
Crude HR	1.00	0.73 (0.55–0.97)	0.88 (0.67–1.16)	0.78 (0.59–1.03)	0.85 (0.65–1.12)	0.380
HR1^a^	1.00	0.76 (0.57–1.01)	0.90 (0.68–1.18)	0.76 (0.57–1.00)	0.81 (0.61–1.06)	*0.160*
HR2^b^	1.00	0.76 (0.57–1.01)	0.91 (0.69–1.20)	0.77 (0.58–1.03)	0.82 (0.62–1.08)	*0.216*
Fruits						
Person-years	153,484	183,463	183,082	183,908	181,753	
Number of cases	99	87	100	85	99	
Crude rate (per 100,000 person-years)	64.50	47.42	54.62	46.22	54.47	
Crude HR	1.00	0.81 (0.61–1.07)	0.93 (0.71–1.22)	0.78 (0.59–1.04)	0.93 (0.70–1.22)	*0.559*
HR1^a^	1.00	0.74 (0.56–0.99)	0.78 (0.59–1.02)	0.62 (0.46–0.83)	0.68 (0.51–0.91)	*0.005*
HR2^b^	1.00	0.75 (0.57–1.01)	0.78 (0.59–1.03)	0.62 (0.46–0.84)	0.69 (0.52–0.92)	*0.006*
Green and yellow vegetables						
Person-years	179,265	182,050	183,736	183,793	184,708	
Number of cases	119	83	79	91	105	
Crude rate (per 100,000 person-years)	66.38	45.59	43.00	49.51	56.85	
Crude HR	1.00	0.68 (0.51–0.90)	0.64 (0.48–0.85)	0.73 (0.56–0.96)	0.83 (0.64–1.09)	*0.293*
HR1^a^	1.00	0.77 (0.58–1.02)	0.69 (0.52–0.91)	0.76 (0.58–1.00)	0.84 (0.64–1.09)	*0.204*
HR2^b^	1.00	0.79 (0.59–1.04)	0.70 (0.52–0.93)	0.79 (0.60–1.04)	0.84 (0.64–1.10)	*0.236*

Abbreviations: HR, hazard ratio; 95% CI, 95% confidence intervals.

^a^HR1: adjusted for age (continuous) and area (11 public health centers).

^b^HR2: adjusted for age (continuous), area (11 public health centers), BMI (14 to <21, 21 to <23, 23 to <25, or 25 to 40 kg/m^2^), smoking status (never, past, current with 1 to 19, or current with ≥20 cigarettes/d), alcohol intake (nondrinker, occasional drinker, <300, or ≥300 g/week in men; nondrinker, occasional drinker, <150, or ≥150 g/week in women), Met’s (quartiles), coffee intake (never, ≤6 cups/week, 1 cup/day, 2 to 3 cups/day, or ≥4 cups/day), green tea intake (ever, ≤6 cups/week, 1 cup/day, 2 to 3 cups/day, or ≥4 cups/day), use of medication for hypertension (yes or no), use of vitamin supplementation (yes or no), postmenopausal status (yes or no; women only), occupation (salaried/self-employed/professional, agriculture/forestry/fishery, housework/unemployed, multiple occupations/others), and total energy intake (continuous).

Table 3 presents the results from the stratified analysis by age. While the main analysis did not show a significant inverse association, the effects of vitamin E, α- and β-carotene intake on pneumonia mortality varied significantly between younger and older participants in men, as indicated by a significant *p*-value for interaction by age stratification (0.030 for vitamin E; 0.001 for α-carotene; 0.001 for β-carotene). Among men older than 60 years, the HR in the fourth quintile of α-carotene intake was 0.64 (95% CI, 0.49–0.85) compared to Q1, with a *p*-trend of 0.068. Conversely, no association was observed among younger participants. Similarly, among men older than 60 years, HRs in the second, third, fourth, and fifth quintiles of β-carotene intake were significantly lower than in Q1, while there appeared to be a trend toward increased risk among younger participants.

**Table 3:** Hazard Ratios for Pneumonia Mortality by Quintiles of Intake of Antioxidant Nutrients Stratified by Age.

	**Men (N = 39,850)**						

	**Q1 (Reference)**	**Q2**	**Q3**	**Q4**	**Q5**	***P*-trend**	***P*-interaction**
Vitamin C							*0.492*
Age ≤60 y							
Person-years	119,610	115,894	107,781	100,770	85,970		
Number of cases	56	55	53	46	30		
HR2^a^	1.00	0.97 (0.66–1.42)	0.98 (0.66–1.46)	0.88 (0.58–1.35)	0.63 (0.39–1.04)	*0.102*	
Age >60 y							
Person-years	25,684	30,732	38,877	45,858	58,561		
Number of cases	78	98	100	129	168		
HR2^a^	1.00	1.09 (0.81–1.48)	0.87 (0.64–1.19)	0.94 (0.69–1.28)	0.94 (0.69–1.29)	*0.499*	
Vitamin E							*0.030*
Age ≤60 y							
Person-years	112,169	113,103	107,272	104,397	93,083		
Number of cases	44.00	52	38.00	50.00	56.00		
HR2^a^	1.00	1.19 (0.79–1.79)	0.89 (0.57–1.39)	1.13 (0.74–1.73)	1.35 (0.88–2.08)	*0.253*	
Age >60 y							
Person-years	32,382	33,762	39,236	42,263	52,071		
Number of cases	117.00	101	85.00	118.00	152.00		
HR2^a^	1.00	0.84 (0.64–1.10)	0.60 (0.45–0.80)	0.80 (0.61–1.05)	0.80 (0.61–1.04)	*0.177*	
α-carotene							*0.001*
Age ≤60 y							
Person-years	110,676	111,801	108,400	105,089	94,059		
Number of cases	42	42	48	62	46		
HR2^a^	1.00	1.03 (0.67–1.59)	1.17 (0.77–1.77)	1.50 (1.00–2.24)	1.18 (0.76–1.84)	*0.128*	
Age >60 y							
Person-years	33,546	34,634	37,820	41,884	51,829		
Number of cases	119	99	118	90	147		
HR2^a^	1.00	0.82 (0.63–1.07)	0.86 (0.67–1.12)	0.64 (0.49–0.85)	0.83 (0.64–1.07)	*0.068*	
β-carotene							*0.001*
Age ≤60 y							
Person-years	115,221	112,797	108,011	102,316	91,680		
Number of cases	37	48	57	52	46		
HR2^a^	1.00	1.34 (0.87–2.06)	1.65 (1.08–2.50)	1.47 (0.95–2.26)	1.38 (0.88–2.17)	*0.151*	
Age >60 y							
Person-years	29,354	32,945	38,895	43,941	54,578		
Number of cases	120	76	104	120	153		
HR2^a^	1.00	0.56 (0.42–0.75)	0.65 (0.50–0.85)	0.68 (0.53–0.89)	0.71 (0.55–0.91)	*0.110*	
Retinol							*0.345*
Age ≤60 y							
Person-years	104,622	102,814	107,487	110,808	104,294		
Number of cases	50	52	39	61	38		
HR2^a^	1.00	1.20 (0.81–1.77)	0.80 (0.52–1.23)	1.24 (0.85–1.81)	0.75 (0.49–1.16)	*0.334*	
Age >60 y							
Person-years	43,098	42,879	38,880	35,752	39,104		
Number of cases	132	115	94	113	119		
HR2^a^	1.00	0.82 (0.64–1.06)	0.77 (0.59–1.01)	0.97 (0.75–1.26)	0.85 (0.66–1.09)	*0.543*	
Cryptoxanthin							*0.050*
Age ≤60 y							
Person-years	113,080	113,452	110,180	104,605	88,707		
Number of cases	55	66	56	36	27		
HR2^a^	1.00	1.19 (0.83–1.71)	1.05 (0.72–1.54)	0.73 (0.48–1.12)	0.67 (0.41–1.08)	*0.020*	
Age >60 y							
Person-years	31,748	34,162	37,592	42,692	53,519		
Number of cases	98	95	103	115	162		
HR2^a^	1.00	0.94 (0.71–1.25)	0.96 (0.72–1.27)	0.90 (0.68–1.20)	0.93 (0.72–1.22)	*0.611*	
Lycopene							*0.162*
Age ≤60 y							
Person-years	105,710	107,309	100,089	109,002	107,915		
Number of cases	57	48	35	57	43		
HR2^a^	1.00	0.93 (0.63–1.37)	0.71 (0.46–1.08)	1.11 (0.76–1.62)	0.81 (0.54–1.21)	*0.606*	
Age >60 y							
Person-years	40,892	38,496	44,245	39,405	36,675		
Number of cases	143	94	133	100	103		
HR2^a^	1.00	0.68 (0.52–0.88)	0.81 (0.64–1.03)	0.75 (0.58–0.98)	0.74 (0.57–0.96)	*0.069*	
Vegetables							*0.489*
Age ≤60 y							
Person-years	113,608	111,736	108,040	103,608	93,033		
Number of cases	47	49	46	54	44		
HR2^a^	1.00	1.06 (0.71–1.59)	1.04 (0.69–1.57)	1.20 (0.80–1.79)	1.03 (0.67–1.57)	*0.684*	
Age >60 y							
Person-years	30,711	33,846	38,008	43,815	53,333		
Number of cases	101	96	105	120	151		
HR2^a^	1.00	0.94 (0.71–1.24)	0.92 (0.70–1.21)	0.90 (0.69–1.18)	0.93 (0.72–1.21)	*0.603*	
Fruits							*0.066*
Age ≤60 y							
Person-years	112,620	110,449	109,104	104,123	93,728		
Number of cases	57	62	48	42	31		
HR2^a^	1.00	1.10 (0.77–1.59)	0.88 (0.60–1.30)	0.81 (0.53–1.22)	0.64 (0.40–1.01)	*0.022*	
Age >60 y							
Person-years	31,761	36,759	38,306	42,518	50,368		
Number of cases	107	94	96	123	153		
HR2^a^	1.00	0.77 (0.58–1.02)	0.79 (0.59–1.05)	0.88 (0.67–1.16)	0.87 (0.67–1.14)	*0.785*	
Green and yellow vegetables							*0.069*
Age ≤60 y							
Person-years	109,883	109,436	108,880	103,403	98,422		
Number of cases	46	50	43	51	50		
HR2^a^	1.00	1.07 (0.72–1.60)	0.92 (0.61–1.40)	1.13 (0.75–1.70)	1.08 (0.71–1.63)	*0.664*	
Age >60 y							
Person-years	34,121	35,742	38,127	43,494	48,229		
Number of cases	134	92	98	122	127		
HR2^a^	1.00	0.67 (0.52–0.88)	0.69 (0.53–0.90)	0.76 (0.59–0.98)	0.69 (0.54–0.89)	*0.029*	

	**Women (N = 46,705)**						

	**Q1 (Reference)**	**Q2**	**Q3**	**Q4**	**Q5**	***P*-trend**	***P-interaction***

Vitamin C							*0.241*
Age ≤60 y							
Person-years	138,524	134,288	128,822	120,169	114,316		
Number of cases	34	14	17	21	15		
HR2^a^	1.00	0.49 (0.26–0.93)	0.68 (0.36–1.25)	0.84 (0.46–1.55)	0.63 (0.31–1.26)	*0.441*	
Age >60 y							
Person-years	44,382	49,311	54,064	62,497	67,180		
Number of cases	68	74	72	81	81		
HR2^a^	1.00	0.92 (0.66–1.29)	0.82 (0.58–1.16)	0.81 (0.57–1.15)	0.74 (0.52–1.07)	*0.087*	
Vitamin E							*0.593*
Age ≤60 y							
Person-years	124,556	128,884	131,503	129,382	121,793		
Number of cases	20	20	23	23	15		
HR2^a^	1.00	0.95 (0.51–1.78)	1.00 (0.55–1.85)	1.03 (0.56–1.89)	0.71 (0.36–1.42)	*0.477*	
Age >60 y							
Person-years	54,437	52,845	52,059	55,467	62,627		
Number of cases	100	74	58	72	72		
HR2^a^	1.00	0.80 (0.59–1.08)	0.66 (0.47–0.91)	0.76 (0.55–1.04)	0.70 (0.51–0.96)	*0.031*	
α-carotene							*0.196*
Age ≤60 y							
Person-years	124,750	132,037	128,906	130,100	120,326		
Number of cases	25	21	26	15	14		
HR2^a^	1.00	0.82 (0.46–1.47)	1.05 (0.60–1.84)	0.63 (0.33–1.21)	0.60 (0.31–1.17)	*0.106*	
Age >60 y							
Person-years	56,030	49,938	54,571	53,402	63,493		
Number of cases	81	73	67	71	84		
HR2^a^	1.00	1.04 (0.75–1.43)	0.89 (0.64–1.23)	1.01 (0.73–1.40)	1.02 (0.75–1.40)	*0.951*	
β-carotene							*0.096*
Age ≤60 y							
Person-years	129,064	132,381	130,347	125,670	118,656		
Number of cases	24	18	26	20	13		
HR2^a^	1.00	0.80 (0.43–1.47)	1.13 (0.64–1.98)	0.86 (0.47–1.58)	0.58 (0.29–1.15)	*0.226*	
Age >60 y							
Person-years	50,715	49,450	52,817	58,688	65,766		
Number of cases	78	79	58	69	92		
HR2^a^	1.00	1.02 (0.74–1.40)	0.70 (0.50–0.99)	0.80 (0.57–1.11)	0.97 (0.71–1.32)	*0.451*	
Retinol							*0.634*
Age ≤60 y							
Person-years	118,364	126,367	128,138	132,727	130,523		
Number of cases	20	14	21	21	25		
HR2^a^	1.00	0.76 (0.38–1.51)	1.22 (0.66–2.28)	1.10 (0.59–2.05)	1.22 (0.67–2.21)	*0.305*	
Age >60 y							
Person-years	66,251	55,447	51,934	50,833	52,968		
Number of cases	87	77	76	68	68		
HR2^a^	1.00	0.99 (0.72–1.35)	1.05 (0.76–1.44)	0.98 (0.71–1.36)	0.94 (0.68–1.30)	*0.716*	
Cryptoxanthin							*0.587*
Age ≤60 y							
Person-years	132,854	134,044	130,911	123,904	114,406		
Number of cases	32	17	20	15	17		
HR2^a^	1.00	0.49 (0.27–0.89)	0.59 (0.33–1.05)	0.46 (0.25–0.87)	0.57 (0.31–1.07)	*0.057*	
Age >60 y							
Person-years	49,036	50,890	53,993	58,901	64,614		
Number of cases	80	58	65	77	96		
HR2^a^	1.00	0.70 (0.50–0.99)	0.67 (0.48–0.93)	0.67 (0.49–0.93)	0.71 (0.52–0.98)	*0.075*	
Lycopene							*0.648*
Age ≤60 y							
Person-years	122,764	130,832	127,746	129,893	124,884		
Number of cases	25	14	20	21	21		
HR2^a^	1.00	0.58 (0.30–1.12)	0.84 (0.46–1.53)	0.81 (0.45–1.46)	0.82 (0.45–1.47)	*0.797*	
Age >60 y							
Person-years	61,738	51,150	53,659	54,674	56,213		
Number of cases	103	70	74	68	61		
HR2^a^	1.00	0.78 (0.58–1.06)	0.77 (0.57–1.05)	0.71 (0.52–0.98)	0.61 (0.44–0.84)	*0.003*	
Vegetables							*0.319*
Age ≤60 y							
Person-years	130,257	132,518	128,349	125,135	119,860		
Number of cases	28	21	18	21	13		
HR2^a^	1.00	0.72 (0.41–1.27)	0.65 (0.36–1.19)	0.79 (0.44–1.40)	0.48 (0.25–0.95)	*0.069*	
Age >60 y							
Person-years	48,349	49,023	53,806	59,392	66,864		
Number of cases	80	60	81	68	87		
HR2^a^	1.00	0.75 (0.54–1.05)	0.98 (0.72–1.34)	0.76 (0.55–1.05)	0.93 (0.68–1.27)	*0.689*	
Fruits							*0.104*
Age ≤60 y							
Person-years	130,018	131,492	127,449	125,554	121,605		
Number of cases	29	10	23	19	20		
HR2^a^	1.00	0.31 (0.15–0.64)	0.71 (0.41–1.25)	0.51 (0.28–0.92)	0.56 (0.31–1.01)	*0.170*	
Age >60 y							
Person-years	51,328	51,972	55,633	58,354	60,148		
Number of cases	77	77	77	66	79		
HR2^a^	1.00	0.93 (0.68–1.29)	0.82 (0.59–1.13)	0.65 (0.46–0.92)	0.73 (0.53–1.02)	*0.013*	
Green and yellow vegetables							*0.954*
Age ≤60 y							
Person-years	125,466	133,504	129,610	125,375	122,164		
Number of cases	26	19	19	17	20		
HR2^a^	1.00	0.71 (0.39–1.29)	0.69 (0.38–1.25)	0.63 (0.34–1.17)	0.72 (0.40–1.31)	*0.254*	
Age >60 y							
Person-years	53,799	48,547	54,126	58,418	62,544		
Number of cases	93	64	60	74	85		
HR2^a^	1.00	0.77 (0.56–1.06)	0.66 (0.48–0.92)	0.79 (0.58–1.08)	0.86 (0.64–1.17)	*0.425*	

Abbreviations: HR, hazard ratio; 95% CI, 95% confidence intervals.

^a^HR2: adjusted for area (11 public health centers), BMI (14 to <21, 21 to <23, 23 to <25, or 25 to 40 kg/m^2^), smoking status (never, past, current with 1 to 19, or current with ≥20 cigarettes/d), alcohol intake (nondrinker, occasional drinker, <300, or ≥300 g/week in men; nondrinker, occasional drinker, <150, or ≥150 g/week in women), Met’s (quartiles), coffee intake (never, ≤6 cups/week, 1 cup/day, 2 to 3 cups/day, or ≥4 cups/day), green tea intake (ever, ≤6 cups/week, 1 cup/day, 2 to 3 cups/day, or ≥4 cups/day), use of medication for hypertension (yes or no), use of vitamin supplementation (yes or no), postmenopausal status (yes or no; women only), occupation (salaried/self-employed/professional, agriculture/forestry/fishery, housework/unemployed, multiple occupations/others), and total energy intake (continuous).

Table 4 presents the results from the stratified analysis by BMI. The effects of vitamin C intake on pneumonia mortality showed significant variation between participants with lower and higher BMI, as indicated by a significant p-value for interaction in both genders (0.009 in men; 0.019 in women). A significant inverse association was observed between vitamin C intake and pneumonia mortality among men with a BMI lower than the median (*p*-trend = 0.021), while no association was observed among men with a BMI higher than the median. A similar pattern was observed in women.

**Table 4:** Hazard Ratios for Pneumonia Mortality by Quintiles of Intake of Antioxidant Nutrients Stratified by BMI.

	**Men (N = 39,850)**						

	**Q1 (Reference)**	**Q2**	**Q3**	**Q4**	**Q5**	***P*-trend**	***P*-interaction**
Vitamin C							*0.009*
BMI ≤ median							
Person-years^a^	70,703	72,974	73,052	71,476	69,044		
Number of cases^a^	80	94	112	93	122		
HR2^b^	1.00	1.05 (0.77–1.44)	1.07 (0.78–1.45)	0.75 (0.54–1.05)	0.77 (0.55–1.08)	*0.021*	
BMI > median							
Person-years^a^	74,591	73,652	73,606	75,153	75,487		
Number of cases^a^	55	59	41	83	76		
HR2^b^	1.00	1.03 (0.71–1.52)	0.60 (0.39–0.93)	1.03 (0.70–1.52)	0.79 (0.52–1.20)	*0.386*	
Vitamin E							*0.075*
BMI ≤ median							
Person-years^a^	76,199	74,070	73,182	68,402	65,395		
Number of cases^a^	110	87	75	113	114		
HR2^b^	1.00	0.75 (0.56–1.00)	0.58 (0.43–0.79)	0.88 (0.66–1.17)	0.72 (0.54–0.97)	*0.182*	
BMI > median							
Person-years^a^	68,352	72,795	73,326	78,258	79,760		
Number of cases^a^	51	66	48	55	94		
HR2^b^	1.00	1.15 (0.79–1.67)	0.74 (0.49–1.11)	0.76 (0.51–1.14)	1.07 (0.73–1.55)	*0.734*	
α-carotene							*0.746*
BMI ≤ median							
Person-years^a^	74,922	73,655	72,665	69,021	66,986		
Number of cases^a^	105	80	103	92	119		
HR2^b^	1.00	0.77 (0.57–1.04)	0.90 (0.68–1.19)	0.80 (0.60–1.07)	0.87 (0.65–1.15)	*0.449*	
BMI > median							
Person-years^a^	69,300	72,780	73,556	77,952	78,902		
Number of cases^a^	56	61	63	60	74		
HR2^b^	1.00	1.05 (0.72–1.52)	1.05 (0.72–1.51)	0.86 (0.59–1.25)	0.93 (0.64–1.34)	*0.404*	
β-carotene							*0.755*
BMI ≤ median							
Person-years^a^	73,418	74,266	72,077	70,331	67,157		
Number of cases^a^	98	71	102	108	120		
HR2^b^	1.00	0.66 (0.49–0.91)	0.85 (0.64–1.13)	0.83 (0.62–1.10)	0.79 (0.60–1.06)	*0.439*	
BMI > median							
Person-years^a^	71,158	71,476	74,829	75,926	79,101		
Number of cases^a^	59	53	59	64	79		
HR2^b^	1.00	0.82 (0.56–1.21)	0.82 (0.57–1.19)	0.77 (0.54–1.11)	0.82 (0.57–1.17)	*0.300*	
Retinol							*0.372*
BMI ≤ median							
Person-years^a^	73,303	70,841	70,445	71,995	70,664		
Number of cases^a^	107	109	76	111	97		
HR2^b^	1.00	1.00 (0.76–1.32)	0.72 (0.53–0.98)	1.11 (0.84–1.46)	0.79 (0.60–1.05)	*0.242*	
BMI > median							
Person-years^a^	74,417	74,853	75,921	74,564	72,734		
Number of cases^a^	75	58	57	63	60		
HR2^b^	1.00	0.79 (0.56–1.12)	0.85 (0.59–1.21)	0.94 (0.67–1.33)	0.86 (0.60–1.21)	*0.703*	
Cryptoxanthin							*0.170*
BMI ≤ median							
Person-years^a^	71,556	72,978	71,791	72,778	68,146		
Number of cases^a^	92	100	105	99	104		
HR2^b^	1.00	1.08 (0.81–1.44)	1.08 (0.81–1.44)	0.87 (0.65–1.17)	0.75 (0.55–1.01)	*0.016*	
BMI > median							
Person-years^a^	73,273	74,636	75,981	74,519	74,080		
Number of cases^a^	61	61	54	52	86		
HR2^b^	1.00	0.94 (0.66–1.36)	0.79 (0.54–1.15)	0.71 (0.48–1.04)	0.97 (0.68–1.40)	*0.562*	
Lycopene							*0.901*
BMI ≤ median							
Person-years^a^	74,019	72,818	71,926	71,071	67,414		
Number of cases^a^	127	86	106	98	82		
HR2^b^	1.00	0.71 (0.53–0.93)	0.81 (0.62–1.05)	0.89 (0.68–1.18)	0.72 (0.54–0.96)	*0.161*	
BMI > median							
Person-years^a^	72,583	72,987	72,408	77,336	77,176		
Number of cases^a^	73	56	62	59	64		
HR2^b^	1.00	0.82 (0.57–1.17)	0.79 (0.55–1.11)	0.83 (0.59–1.19)	0.85 (0.60–1.20)	*0.412*	
Vegetables							*0.171*
BMI ≤ median							
Person-years^a^	75,260	72,873	70,940	70,260	67,914		
Number of cases^a^	88	89	100	115	107		
HR2^b^	1.00	1.03 (0.76–1.39)	1.06 (0.79–1.42)	1.06 (0.80–1.41)	0.89 (0.66–1.20)	*0.501*	
BMI > median							
Person-years^a^	69,058	72,708	75,108	77,162	78,453		
Number of cases^a^	60	56	51	59	88		
HR2^b^	1.00	0.83 (0.57–1.20)	0.68 (0.46–1.00)	0.71 (0.49–1.03)	0.89 (0.63–1.26)	*0.499*	
Fruits							*0.709*
BMI ≤ median							
Person-years^a^	70,900	72,350	72,683	71,902	69,413		
Number of cases^a^	106	95	92	100	106		
HR2^b^	1.00	0.82 (0.62–1.09)	0.79 (0.59–1.06)	0.77 (0.57–1.02)	0.69 (0.51–0.93)	*0.019*	
BMI > median							
Person-years^a^	73,481	74,859	74,728	74,739	74,683		
Number of cases^a^	58	61	52	66	78		
HR2^b^	1.00	0.97 (0.67–1.39)	0.79 (0.53–1.16)	0.92 (0.63–1.34)	0.94 (0.64–1.37)	*0.748*	
Green and yellow vegetables							*0.111*
BMI ≤ median							
Person-years^a^	72,750	73,226	72,232	70,287	68,753		
Number of cases^a^	102	91	91	117	98		
HR2^b^	1.00	0.88 (0.66–1.17)	0.84 (0.63–1.12)	1.00 (0.76–1.32)	0.76 (0.57–1.01)	*0.205*	
BMI > median							
Person-years^a^	71,255	71,952	74,775	76,610	77,898		
Number of cases^a^	78	51	50	56	79		
HR2^b^	1.00	0.66 (0.46–0.94)	0.58 (0.41–0.83)	0.59 (0.41–0.84)	0.74 (0.53–1.03)	*0.075*	

	**Women (N = 46,705)**						

	**Q1 (Reference)**	**Q2**	**Q3**	**Q4**	**Q5**	***P*-trend**	***P*-interaction**

Vitamin C							*0.019*
BMI ≤ median							
Person-years^a^	88,016	91,285	89,502	91,150	89,797		
Number of cases^a^	60	36	54	58	60		
HR2^b^	1.00	0.55 (0.36–0.84)	0.81 (0.55–1.21)	0.73 (0.49–1.10)	0.71 (0.47–1.08)	*0.403*	
BMI > median							
Person-years^a^	94,890	92,313	93,384	91,516	91,699		
Number of cases^a^	42	52	35	44	36		
HR2^b^	1.00	1.20 (0.78–1.83)	0.74 (0.46–1.19)	0.93 (0.59–1.48)	0.67 (0.40–1.11)	*0.064*	
Vitamin E							*0.303*
BMI ≤ median							
Person-years^a^	91,680	90,624	91,536	91,848	84,062		
Number of cases^a^	67	46	44	57	53		
HR2^b^	1.00	0.74 (0.50–1.08)	0.73 (0.49–1.08)	0.89 (0.62–1.29)	0.78 (0.53–1.15)	*0.447*	
BMI > median							
Person-years^a^	87,313	91,105	92,026	93,001	100,358		
Number of cases^a^	53	48	37	38	34		
HR2^b^	1.00	1.02 (0.68–1.52)	0.78 (0.50–1.21)	0.79 (0.51–1.23)	0.62 (0.39–0.99)	*0.023*	
α-carotene							*0.434*
BMI ≤ median							
Person-years^a^	91,705	91,804	90,843	89,688	85,709		
Number of cases^a^	53	54	60	49	52		
HR2^b^	1.00	1.22 (0.82–1.80)	1.29 (0.89–1.89)	1.12 (0.75–1.67)	1.01 (0.67–1.51)	*0.929*	
BMI > median							
Person-years^a^	89,074	90,171	92,635	93,813	98,110		
Number of cases^a^	53	40	33	37	46		
HR2^b^	1.00	0.92 (0.60–1.41)	0.74 (0.48–1.17)	0.86 (0.56–1.32)	0.96 (0.63–1.47)	*0.722*	
β-carotene							*0.780*
BMI ≤ median							
Person-years^a^	90,570	90,626	90,897	91,068	86,590		
Number of cases^a^	58	48	49	54	60		
HR2^b^	1.00	0.91 (0.61–1.34)	0.87 (0.59–1.29)	0.90 (0.61–1.32)	0.88 (0.60–1.28)	*0.526*	
BMI > median							
Person-years^a^	89,209	91,205	92,267	93,290	97,832		
Number of cases^a^	44	49	35	35	45		
HR2^b^	1.00	1.14 (0.75–1.73)	0.85 (0.54–1.34)	0.82 (0.51–1.30)	0.90 (0.58–1.40)	*0.302*	
Retinol							*0.261*
BMI ≤ median							
Person-years^a^	86,776	92,925	91,973	90,806	87,271		
Number of cases^a^	62	50	48	53	56		
HR2^b^	1.00	0.89 (0.61–1.31)	0.90 (0.61–1.33)	1.01 (0.69–1.48)	1.08 (0.74–1.57)	*0.561*	
BMI > median							
Person-years^a^	97,840	88,889	88,099	92,754	96,220		
Number of cases^a^	45	41	49	37	37		
HR2^b^	1.00	1.11 (0.72–1.73)	1.51 (0.99–2.30)	1.10 (0.70–1.73)	0.98 (0.62–1.56)	*0.991*	
Cryptoxanthin							*0.474*
BMI ≤ median							
Person-years^a^	87,708	91,930	91,202	91,092	87,818		
Number of cases^a^	67	46	50	48	58		
HR2^b^	1.00	0.66 (0.45–0.96)	0.65 (0.44–0.95)	0.56 (0.38–0.82)	0.60 (0.42–0.88)	*0.007*	
BMI > median							
Person-years^a^	94,182	93,004	93,702	91,713	91,202		
Number of cases^a^	45	30	35	44	55		
HR2^b^	1.00	0.69 (0.43–1.11)	0.69 (0.43–1.09)	0.77 (0.50–1.20)	0.82 (0.53–1.25)	*0.585*	
Lycopene							*0.867*
BMI ≤ median							
Person-years^a^	85,644	89,679	94,109	93,011	87,307		
Number of cases^a^	74	46	56	50	43		
HR2^b^	1.00	0.72 (0.49–1.05)	0.81 (0.57–1.16)	0.70 (0.48–1.01)	0.62 (0.42–0.91)	*0.019*	
BMI > median							
Person-years^a^	98,858	92,303	87,295	91,556	93,790		
Number of cases^a^	54	38	38	40	39		
HR2^b^	1.00	0.89 (0.58–1.35)	0.81 (0.53–1.25)	0.85 (0.56–1.30)	0.79 (0.52–1.21)	*0.288*	
Vegetables							*0.111*
BMI ≤ median							
Person-years^a^	91,378	92,300	89,617	89,407	87,048		
Number of cases^a^	52	43	56	54	64		
HR2^b^	1.00	0.83 (0.55–1.25)	1.08 (0.73–1.59)	0.98 (0.66–1.45)	1.09 (0.74–1.60)	*0.471*	
BMI > median							
Person-years^a^	87,228	89,241	92,537	95,120	99,676		
Number of cases^a^	56	38	43	35	36		
HR2^b^	1.00	0.69 (0.45–1.05)	0.75 (0.50–1.13)	0.58 (0.37–0.89)	0.56 (0.36–0.87)	*0.007*	
Fruits							*0.126*
BMI ≤ median							
Person-years^a^	99,676	88,834	92,219	91,829	90,758		
Number of cases^a^	62	58	55	42	52		
HR2^b^	1.00	0.78 (0.54–1.13)	0.66 (0.45–0.97)	0.48 (0.32–0.72)	0.58 (0.39–0.85)	*0.001*	
BMI > median							
Person-years^a^	95,236	94,629	90,863	92,078	90,995		
Number of cases^a^	44	29	45	43	48		
HR2^b^	1.00	0.65 (0.40–1.06)	0.94 (0.60–1.47)	0.84 (0.54–1.31)	0.84 (0.54–1.31)	*0.792*	
Green and yellow vegetables							*0.341*
BMI ≤ median							
Person-years^a^	88,521	90,554	91,181	90,029	89,465		
Number of cases^a^	68	42	48	45	65		
HR2^b^	1.00	0.71 (0.48–1.05)	0.75 (0.51–1.09)	0.67 (0.45–0.99)	0.88 (0.62–1.26)	*0.463*	
BMI > median							
Person-years^a^	90,744	91,496	92,556	93,764	95,243		
Number of cases^a^	51	41	31	46	40		
HR2^b^	1.00	0.90 (0.59–1.37)	0.62 (0.40–0.98)	0.92 (0.61–1.40)	0.77 (0.49–1.19)	*0.314*	

Abbreviations: BMI, body mass index; HR, hazard ratio; 95% CI, 95% confidence intervals.

^a^Person-years and number of cases are presented as the mean values pooled across 20 imputated datasets.

^b^HR2: adjusted for age (continuous), area (11 public health centers), smoking status (never, past, current with 1 to 19, or current with ≥20 cigarettes/d), alcohol intake (nondrinker, occasional drinker, <300, or ≥300 g/week in men; nondrinker, occasional drinker, <150, or ≥150 g/week in women), Met’s (quartiles), coffee intake (never, ≤6 cups/week, 1 cup/day, 2 to 3 cups/day, or ≥4 cups/day), green tea intake (ever, ≤6 cups/week, 1 cup/day, 2 to 3 cups/day, or ≥4 cups/day), use of medication for hypertension (yes or no), use of vitamin supplementation (yes or no), postmenopausal status (yes or no; women only), occupation (salaried/self-employed/professional, agriculture/forestry/fishery, housework/unemployed, multiple occupations/others), and total energy intake (continuous).

Based on the results of the stratified analysis by smoking status, the associations between antioxidant nutrients, vegetables, and fruits intake and pneumonia mortality risk did not significantly differ by smoking status in either gender (as shown in Supplementary Table 7).

## Discussion

This population-based prospective cohort study provides evidence from a Japanese population that certain antioxidant nutrients, particularly cryptoxanthin, lycopene, vitamin C, and vitamin E, are associated with a reduced risk of pneumonia mortality. Although the univariable model initially suggested that higher intakes of vitamin C, vitamin E, and cryptoxanthin were associated with an increased risk of pneumonia mortality among men, inverse associations were observed after adjustment for potential confounding factors. This discrepancy reflects substantial confounding by demographic and lifestyle factors. After controlling for these confounders, the inverse associations became evident, suggesting that these nutrients may play a protective role in pneumonia. These findings remained robust even after excluding the early deaths that occurred within the first 3 years of follow-up or participants who used vitamin supplements.

The protective effect of these antioxidant nutrients may be attributed to their common underlying mechanism of reducing oxidative stress and inflammation in pulmonary tissues. By neutralising reactive oxygen species, these nutrients help to combat oxidative stress, a key factor in the pathophysiology of pneumonia, thereby reducing inflammation and tissue damage in the lungs [13, 36–39]. They also support immune system function, maintain epithelial barrier function, modulate pro-inflammatory cytokine levels, and enhance antioxidant enzyme activities [4, 13, 36, 39–46]. These collective actions contribute to a reduction in pneumonia severity and mortality.

Our findings on antioxidant nutrients align partially with previous research. A review reported the positive inter-relationship between cryptoxanthin and pulmonary performance, indirectly suggesting a beneficial effect on respiratory health and potentially lower pneumonia mortality [47]. Higher serum levels of lycopene and vitamin C have been associated with reduced mortality from influenza or pneumonia in the US population [4, 11, 19]. Notably, our study found that the impact of vitamin C intake on pneumonia mortality varied significantly with BMI. A significant inverse association was observed among participants with a BMI below the median but not among those with higher BMI, a pattern consistent in both men and women. This suggests that BMI may modify the association between vitamin C and pneumonia mortality. A previous study has reported a negative correlation between vitamin C concentration and BMI, suggesting that individuals with higher BMI may require greater vitamin C intake to achieve protective effects [48]. Further research is needed to investigate the effect of vitamin C on pneumonia while accounting for BMI differences. Several studies have investigated the association between vitamin E supplementation and pneumonia; they showed controversial results. Some suggest that vitamin E may increase pneumonia risk in specific subgroups, such as heavy smokers or individuals with high dietary vitamin C intake [8, 9, 23, 49]. Others report potential benefits in chronic lung diseases like chronic obstructive pulmonary disease, indicating that it may reduce respiratory disease risk [7]. These inconsistent findings may be due to differences in study populations, dosages, and interactions with other nutrients and lifestyle factors. In addition, our age-stratified analysis revealed a significant interaction between vitamin E, α-carotene, β-carotene intake and age in men, which suggests that these nutrients tended to be associated with reduced pneumonia mortality in participants older than 60 years, whereas in younger participants the effect may even adverse. These findings align with results from the Alpha-Tocopherol Beta-Carotene Cancer Prevention (ATBC) Study, which reported that vitamin E showed a protective effect among older participants, whereas β-carotene increased the risk of pneumonia in younger smokers [24]. Older adults, who are subject to increasing oxidative stress and immune senescence, may enhance their sensitivity to antioxidants. In contrast, younger adults with more resilient physiology who consume antioxidants beyond adequate levels may not experience additional benefits and may instead experience adverse effects. Furthermore, residual confounding from factors such as smoking or alcohol consumption in younger adults could also partly explain these differential findings. Retinol was the only nutrient that showed no significant association with pneumonia mortality in either overall or stratified analyses. One possible explanation for this null finding may relate to the relatively low dietary retinol intake in our Japanese cohort. In our study, median dietary retinol intake among Japanese men ranged from 93.2 mcg/day in the lowest quintile to 1081.3 mcg/day in the highest quintile. In contrast, a previous cohort study conducted within the ATBC Study of Finnish male smokers reported a significant inverse association between serum retinol concentrations and respiratory disease mortality. Although serum retinol concentrations and dietary retinol intake do not always correspond directly, the dietary retinol intake observed in the highest quintile of our cohort was substantially lower than the mean dietary vitamin A intake (1780 mcg/day) reported in the lowest quintile of the ATBC Study [50]. Therefore, the relatively low dietary retinol levels in our study might partially explain the lack of a clear protective association with pneumonia mortality. It is important to note that the ATBC study participants were exclusively Finnish male smokers who subsequently underwent β-carotene and vitamin E interventions, indicating that the unique characteristics of this population should be considered carefully when comparing results with our general Japanese population.

While our study primarily focused on individual antioxidant nutrients, we also investigated the intake of vegetables and fruits, which are primary dietary sources of these antioxidants. In our results, fruits showed a significant association with reduced pneumonia mortality, which was significant in women and marginally not significant in men. Vegetables showed no association with pneumonia mortality. These findings are similar to those of another earlier JPHC study by Sahashi et al. [34], which also found that fruits consumption among men was associated with a reduced risk of death from respiratory disease. The study by Sahashi et al. examined overall respiratory disease mortality, whereas our study specifically focused on pneumonia mortality. The consistency in results between our study and Sahashi et al.’s suggests that fruits may have a protective effect against pneumonia mortality in the Japanese population, possibly due to their high content of cryptoxanthin and lycopene, which are more abundant in fruits than in vegetables.

The present study has several strengths, including a large general study population, a long-term follow-up of over 20 years and inclusion of over 1000 cases of pneumonia death, a high response rate, and a prospective design. However, several limitations should also be mentioned. First, the consumption of antioxidant nutrients, vegetables and fruits, as well as other dietary and lifestyle factors, was assessed at a single point using a self-administered FFQ questionnaire with moderate validity coefficients. Consequently, exposure misclassification and changes in these behaviors over time may have affect the risk of pneumonia mortality. Future studies that consider changes in dietary and lifestyle and evaluate their association with pneumonia mortality are needed. Second, the generalizability of the study was somewhat limited because the participants included in the analysis were relatively healthy adults. Third, we were unable to adjust for certain important potential confounders, including education level, history of chronic obstructive pulmonary disease, and passive smoking, because these data were not available. Additionally, residual confounding due to smoking is likely present. Our analysis was unable to accurately account for cumulative smoking exposure, as the 5-year survey lacked information on age at smoking initiation and cessation, preventing us from calculating of precise pack-years. Although we partially addressed smoking intensity among current smokers by considering daily cigarette consumption, incomplete assessment of cumulative smoking exposure could still have resulted in residual confounding. Fourth, dietary nutrient intake was calculated using the seventh edition of the Standard Tables of Food Composition in Japan. Given that the eighth edition has subsequently been published, some nutrient values might have changed, which could potentially lead to minor misclassification of dietary intake.

In conclusion, this study suggests that increased dietary intake of antioxidant nutrients may be associated with a reduced risk of pneumonia mortality in the Japanese middle-aged population.

## Supplementary information

**Additional file 1:** **Supplementary Table 1.** Basic Characteristics by Quintiles of Dietary Intake of Antioxidant Nutrients, Vegetables, and Fruits in Japanese Men and Women. **Supplementary Table 2.** Hazard Ratios for Pneumonia Mortality by Quintiles of Intake of Antioxidant Nutrients, Vegetables, and Fruits in Japanese Men and Women (Sensitivity Analysis: Exclusion of Participants Who Died within the First Three Years of Follow-up). **Supplementary Table 3.** Hazard Ratios for Pneumonia Mortality by Quintiles of Intake of Nutrients, Vegetables, and Fruits in Japanese Men and Women (Sensitivity Analysis: Exclusion of Participants Who Used Vitamin Supplements). **Supplementary Table 4.** Hazard Ratios for Pneumonia Mortality by Quintiles of Intake of Antioxidant Nutrients, Vegetables, and Fruits in Japanese Men and Women (Complete Case Analysis: Main Analysis). **Supplementary Table 5.** Hazard Ratios for Pneumonia Mortality by Quintiles of Intake of Antioxidant Nutrients, Vegetables, and Fruits in Japanese Men and Women (Complete Case Analysis: Exclusion of Participants Who Died within the First Three Years of Follow-up). **Supplementary Table 6.** Hazard Ratios for Pneumonia Mortality by Quintiles of Intake of Antioxidant Nutrients, Vegetables, and Fruits in Japanese Men and Women (Complete Case Analysis: Exclusion of Participants Who Used Vitamin Supplements). **Supplementary Table 7.** Hazard Ratios for Pneumonia Mortality by Quintiles of Intake of Antioxidant Nutrients, Vegetables, and Fruits, Stratified by Smoking Status in Japanese Men and Women.

## Declarations

### Availability of data and material

For information on data availability, please refer to this link: https://epi.ncc.go.jp/en/jphc/805/8155.html.
